# Plant growth promoting rhizobacteria alleviates drought stress in potato in response to suppressive oxidative stress and antioxidant enzymes activities

**DOI:** 10.1038/s41598-020-73489-z

**Published:** 2020-10-12

**Authors:** Tahira Batool, Shafaqat Ali, Mahmoud F. Seleiman, Naima Huma Naveed, Aamir Ali, Khurshid Ahmed, Muhammad Abid, Muhammad Rizwan, Muhammad Rizwan Shahid, Majed Alotaibi, Ibrahim Al-Ashkar, Muhammad Mubushar

**Affiliations:** 1 Department of Botanical Sciences, University of Sargodha, Punjab, 40210 Pakistan; 2 Department of Environment Sciences and Engineering, Government College University, Faisalabad, 38000 Pakistan; 3 Plant Production Department, College of Food and Agriculture Sciences, King Saud University, P.O. Box 2460, Riyadh, 11451 Saudi Arabia; 4 Department of Crop Sciences, Faculty of Agriculture, Menoufia University, Shibin El-kom, 32514 Egypt; 5 Key Laboratory of Crop Physiology, Ecology and Production Management, Nanjing Agricultural University, Nanjing, 210095 Jiangsu People’s Republic of China; 6 Agriculture Department (Field Wing), Punjab, Pakistan; 7 Institute of Soil and Environmental Sciences, University of Agriculture, Faisalabad, 38000 Pakistan; 8 Agronomy Department, Faculty of Agriculture, Al-Azhar University, Cairo, 11651 Egypt

**Keywords:** Plant signalling, Plant signalling, Plant signalling, Plant signalling, Drought

## Abstract

Maintenance of plant physiological functions under drought stress is normally considered a positive feature as it indicates sustained plant health and growth. This study was conducted to investigate whether plant growth-promoting rhizobacteria (PGPR) *Bacillus subtilis* HAS31 has potential to maintain potato growth and yield under drought stress. We analyzed trends of chlorophyll concentration, photosynthesis process, relative water content, osmolytes, antioxidants enzymes and oxidative stress, relative growth rate, tuber and aboveground biomass production in two potato varieties, Santae (drought-tolerant) and PRI-Red (drought-sensitive). Plants of both genotypes were treated with 100 g of HAS31 inoculant at 10 days after germination and exposed to different soil relative water contents (SRWC), including 80 ± 5% (well watered), 60 ± 5% (moderate stress) and 40 ± 5% SRWC (severe stress) for 7 days at tuber initiation stage (30 days after germination). The drought stress reduced plant relative growth rate, biomass production, leaf area, number of leaves and tubers, tuber weight, and final yield. The drought-stressed plants showed decline in chlorophyll contents, membrane stability, leaf relative water contents and photosynthetic rate. Under drought stress, enzymatic activity of catalase (CAT), peroxidase (POD) and superoxide dismutase (SOD), contents of total soluble sugars, soluble proteins and proline increased. The application of PGPR reduced the impact of drought and maintained higher growth and physio-chemical traits of the plants. The plants with PGPR application showed higher relative growth rate, dry matter production, leaf area, number of tubers, tuber weight and yield as compared to plants without PGPR. The PGPR-HAS31 treated plants maintained higher photosynthetic process, contents of chlorophyll, soluble proteins, total soluble sugars, and enzymatic activities of CAT, POD and SOD as compared to plants without PGPR. The results of the study suggest that plant growth regulators have ability to sustain growth and yield of potato under drought stress by maintaining physiological functions of the plants.

## Introduction

Potato (*Solanum tuberosum* L.) is the largest vegetable crop which is grown in 79% countries of the world^1^. It stands at fourth position in production volume after rice, wheat and maize in the world and provides the cheapest and easy source of proteins, essential amino acids, carbohydrates, minerals, antioxidants and vitamins around the globe^2–4^. To ensure food availability to the rapidly growing world population that is estimated to reach about six billion by the end of year 2050^5^, a substantial increase in potato production is required^6^. Despondently, declining water resources challenge this perception, as a large area under potato cultivation is affected by periodic drought events^7^. Many areas of potato production in developing countries are located in semi-arid areas, where drought spells account for large harvest losses and thus threaten sustainability of the agricultural industry^8,9^.

As compared to other crops, potato is more sensitive to drought stress. Drought occurrence, especially during early tuberization affects yield by decreasing both number and weight of tubers^10^. reported a significant decrease in tuber yield due to drought stress applied during tuber bulking stage, but effects of drought were variable due to differences in genotypes, drought levels and growth stages of the crop. Water deficiency, especially during the tuber bulking period decreases yield to larger extents than water limitation during other growth stages of potato^11^.

Crop yield is the end product of photosynthesis and other interlinked physiological processes. Lower plant growth and productivity due to drought are mainly caused by altered plant water relations, decreased photosynthetic process, cellular-oxidative load, membrane damage, and in some instances, inhibited enzyme activity. Oscillations in the normal values of the physiological functions are the indicators of plant health and environmental conditions available to the plant^12^. It is admitted that mild drought stress reduces the rate of net photosynthesis due to stomatal limitation; meanwhile, the photosynthetic apparatus is not significantly affected. In disparately, under severe drought conditions, stomatal limitation plus reduced efficiency of photosystem II (PSII) and poor activities of CO_2_ assimilating enzymes become the primary restrictions to diminished photosynthetic rates^13,14^.

To ensure a sustainable potato yield in an agro-system with ever increasing environmental stresses, the plant breeders are challenged to develop varieties having potential genetic makeup for high yield and resistance against environmental stresses^15^. So far, by conventional breeding and advanced biotechnological and transgenic approaches an auspicious contribution has been made to drought tolerance development in crop plants. Due to restricted genetic biodiversity and ecological limitations, the further development in such advanced expertise may be limited to get increased production of crops^16^. However, crop management approaches aimed at improving tolerance to adversative conditions can offer a great potential to alleviate effects and yield losses under stress conditions^17,18^.

Plant growth-promoting rhizobacteria (PGPR) are naturally occurring soil bacteria and are known to induce plant growth promotion. The favorable effects of PGPR have been reported in many crop species^19–22^. The benefits of plant–PGPR interactions have been reported in improved seed germination, root development, shoot and root weights, leaf area, chlorophyll content, hydraulic activity, protein content, nutrient uptake, and yields in the previous studies in corn, wheat, vegetables, potato and cassava^23–27^.

There is also evidence that beneficial microbes can enhance plants’ tolerance against adverse environmental conditions. For example, Arbuscular mycorrhizal fungi enhanced salinity-tolerance of *Panicum turgidum* Forssk by favorably altering photosynthetic and antioxidant pathways^28^. Due to application of potassium-releasing strain of *Bacillus edaphicus* an enhanced plant growth and potassium uptake have been reported under nutrient deficiency and heavy metal contamination in cotton and rapeseeds^29^. Similarly, the plants of tomato, okra, and African spinach treated with PGPR (*Rhizobium, Azospirillum, Pseudomonas, Flavobacterium, Arthrobacter* and *Bacillus*) displayed their improved osmoregulation, oligotrophic, endogenous metabolism, resistance to starvation, and thus showed their efficient metabolic processes to adapt dry and saline environments^29,30^. However, the evidence whether PGPR isolated as *Bacillus subtilis* HAS31 has ability to induce tolerance against drought stress in potato, is not clear yet.

The present research work was carried out to investigate whether application of growth promoting substances has potential to sustain plant growth and yield of potato by regulating physiological, biochemical and agronomic traits under drought stress.

## Results

Two potato cultivars, namely Santae and PRI-Red referred to as ‘tolerant’ and ‘sensitive’, respectively were exposed to a range of water levels, including well-watered, moderate and severe drought stresses during tuber initiation stage followed by re-watering to quantify the agronomic, physiological and metabolic changes in the condition of plants with plant growth promoting rhizobacteria (PGPR) and without PGPR applications.

### Changes in relative growth rate and above-ground dry matter production

The drought stress applied during tuberization stage in the present study resulted in a significant reduction in relative growth rate (RGR) and plant dry matter production in both cultivars (Table 1). Under well watered conditions, the RGR was not significantly different in both cultivars; however, variations were noted among the treatments for these traits under stress and re-watered conditions as well. The plants with PGPR displayed less decrease in RGR and dry matter due to drought stress than the plants without PGPR application. For example, during the drought period, RGR decreased by 42 and 88% in plants with PGPR and by 51 and 95% in plants without PGPR in Santae; whereas, the plants of PRI-Red with PGPR showed a decrease in RGR by 61 and 95% and without PGPR the decrease in RGR was up to 65 and 96% under moderate and severe stresses as compared to well watered (CK) conditions, respectively. However, after rewatering, a gradual increase in RGR was observed in drought-stressed plants during the subsequent growth periods of crop, like tuber initiation to tuber bulking and tuber bulking to maturation.

**Table 1:** Effect of drought stress and PGPR application on relative growth rate and dry matter production in two potato cultivars.

Cultivars	PGPR treatments	Drought treatments	Relative growth rate (mg g^−1^ day^−1^)			Dry matter (g/pot)	Pre-drought limitation (%)
			During stress (tuber initiation)	Tuber initiation-tuber bulking	Tuber bulking-maturation		
Santae	With PGPR	CK	28.5 ± 2 a	23.4 ± 1.3 bc	18.5 ± 2 a	65.3 ± 2.6 a	0.00
		MS	16.5 ± 1 c	27.5 ± 1.1 a	16.5 ± 1 b	50.5 ± 2.2 c	22.7 ± 2.2 c
		SS	03.3 ± 3 e	12.7 ± .3 d	11.3 ± 3 d	34.6 ± 2.4 e	45.0 ± 2.4 b
	Without PGPR	CK	27.6 ± 1.2 ab	21.3 ± .5 c	16.6 ± 1.2 b	62.3 ± 2 ab	0.00
		MS	12.6 ± 1.5 d	22.3 ± 1 c	12.6 ± 1.5 c	44.5 ± 3 d	24.5 ± 3 d
		SS	1.3 ± 0.5 f.	10.3 ± .2 e	8.3 ± 0.5 e	31.6 ± 1.2 f.	50.6 ± 1.2 a
PRI-Red	With PGPR	CK	31.4 ± 1 a	24.4 ± 1.2 a	19.4 ± 1 a	64.5 ± 1 a	0.00
		MS	12.5 ± 2 c	23.3 ± 1.2 ab	15.5 ± 2 c	49.6 ± 2 c	25.6 ± 2 c
		SS	1.5 ± 0.5 e	9.2 ± 1.5 c	7.5 ± 0.5 e	25.3 ± 1 e	60.3 ± 1 b
	Without PGPR	CK	30.5 ± 1 ab	22.2 ± 1 b	17.5 ± 1 b	60.4 ± 3 b	0.00
		MS	10.6 ± 0.6 d	24.1 ± 1 a	12.6 ± 0.6 d	42 ± 2.6 d	30 ± 2.6 d
		SS	1.0 ± 0.5 f	7.5 ± .2 d	5.5 ± 0.5 f.	22 ± 1.4 f.	63.3 ± 1.4 a

Plants of both genotypes were treated with 100 g of bio-fertilizer at 10 days after germination and exposed to different soil relative water contents (SRWC), including 80 ± 5% (well watered; CK), 60 ± 5% (moderate stress; MS) and 40 ± 5% SRWC (severe stress; SS) for 7 days at tuber initiation stage (30 days after germination). Lowercase letter after each data in the column indicates significant difference at P < 0.05 between the treatments for each cultivar separately.

Similarly, above-ground dry matter production was decreased in both cultivars due to drought stress (Table 1). Under well watered conditions, the dry matter was not significantly different in both cultivars. However, in drought stressed treatments, the plants with PGPR displayed less decrease in dry matter due to the drought stress than the plants without PGPR application. In Santae, dry matter decreased by 22 and 46% in plants with PGPR and by 31 and 55% in plants without PGPR; whereas, the plants of PRI-Red with PGPR showed a decrease in dry matter by 24 and 60%, but without PGPR the decrease in dry matter was up to 28 and 61% under moderate and severe droughts as compared to well watered conditions, respectively. However, the plants of Santae both with and without PGPR showed less decrease in dry matter than the plants of PRI-Red.

Pre-drought limitation to dry matter production at maturity increased as the level of drought increased (Table 1). However, the plants of both cultivars with PGPR showed less pre-drought limitation than the plants without PGPR. The plants of Santae both with and without PGPR showed less pre-drought limitation than the plants of PRI-Red. In Santae, the values of pre-drought limitation were as 22 and 45% under PGPR and 24 and 50% without PGPR; whereas pre-drought limitation in PRI-Red was 25 and 60% with PGPR and 30 and 63% without PGPR, respectively.

### Changes in leaf area traits

The drought treatments resulted in a significant reduction in leaf area per plant in both cultivars (Table 2). The plants with PGPR treatments showed higher leaf areas as compared to plants without PGPR both under normal and stressed plant conditions. The plants of Santae with and without PGPR maintained higher leaf areas than the plants of PRI-Red. The leaf area per plant decreased by 18 and 68% in plants with PGPR and by 22 and 80% in plants without PGPR in Santae; whereas, the plants of PRI-Red with PGPR showed a decrease in leaf area by 29 and 75%, while without PGPR the decrease in RGR was up to 41 and 78% under moderate and severe drought stresses, respectively as compared to well watered conditions.

**Table 2:** Effect of drought stress and PGPR application on leaf area, specific leaf area, leaf dry matter content and number of leaves per plant in two potato cultivars.

Cultivars	PGPR Treatments	Drought treatments	Leaf area/plant (cm^2^)	Specific leaf area (cm^2^/g)	Leaf dry matter content (mg/g)	Number of leaves/plant
Santae	With PGPR	CK	42.3 ± 2 a	245.1 ± 8 a	300.3 ± 14 d	20.3 ± 1.4 a
		MS	36.5 ± 1 c	200.5 ± 6 c	317.5 ± 15 b	17.5 ± 1.5 b
		SS	13.3 ± 3 e	130.3 ± 7 e	332.7 ± 13 a	12.7 ± .3 c
	Without PGPR	CK	37.6 ± 1.2 b	205.3 ± 9 b	307.3 ± 10 c	17.3 ± 5 b
		MS	30.6 ± 1.5 d	150.3 ± 11 d	316.3 ± 12 b	16.3 ± 2 c
		SS	7.3 ± 0.5 f.	100.7 ± 6 f.	330.3 ± 12 a	10.3 ± 2 d
PRI-Red	With PGPR	CK	45.4 ± 1 a	242.3 ± 10 b	317.4 ± 12 f.	17.4 ± 1.2 a
		MS	32.5 ± 2 b	180.3 ± 8 c	336.3 ± 12 c	16.3 ± 2 b
		SS	11.5 ± 0.5 d	119 ± 5 e	351.2 ± 15 b	11.2 ± 1.5 c
	Without PGPR	CK	44.5 ± 1 a	250.3 ± 3 a	327.2 ± 13 d	17.2 ± 1 a
		MS	23.6 ± 0.6 c	152.4 ± 7 d	339.1 ± 12 c	16.1 ± 1 b
		SS	9.4 ± 0.5 e	100.6 ± 5 f	360 ± 8 a	10 ± 2 c

Plants of both genotypes were treated with 100 g of bio-fertilizer at 10 days after germination and exposed to different soil relative water contents (SRWC), including 80 ± 5% (well watered; CK), 60 ± 5% (moderate stress; MS) and 40 ± 5% SRWC (severe stress; SS) for 7 days at tuber initiation stage (30 days after germination). Lowercase letter after each data in the column indicates significant difference at P < 0.05 between the treatments for each cultivar separately.

Similarly, specific leaf area was decreased in both cultivars due to drought stress (Table 2). Under well watered conditions, the specific leaf area was not significantly different in both cultivars; however, PGPR application increased specific leaf area. In drought treatments, the plants with PGPR displayed less decrease in specific leaf area due to drought stress than the plants without PGPR application. In Santae, specific leaf area decreased by 19 and 44% in plants with PGPR and by 27 and 57% in plants without PGPR; whereas, the plants of PRI-Red with PGPR showed decrease in specific leaf area by 28 and 56% and without PGPR the specific leaf area decreased up to 35 and 60% under moderate and severe drought stresses, respectively as compared to control conditions.

Leaf dry matter content (LDMC) increased due to drought stress in both cultivars (Table 2). LDMC increased as the level of drought stress increased. However, there was not a significant difference in leaf dry matter content of both cultivars and with and without PGPR application. In Santae, LDMC were increased by 21 and 30% under PGPR and 17 and 25% without PGPR; whereas LDMC in PRI-Red increased by 18 and 26% with PGPR and by 13 and 16% without PGPR under moderate and severe drought stresses, respectively as compared to control conditions.

### Changes in tuber yield per plant and drought index

**Figure 1:**
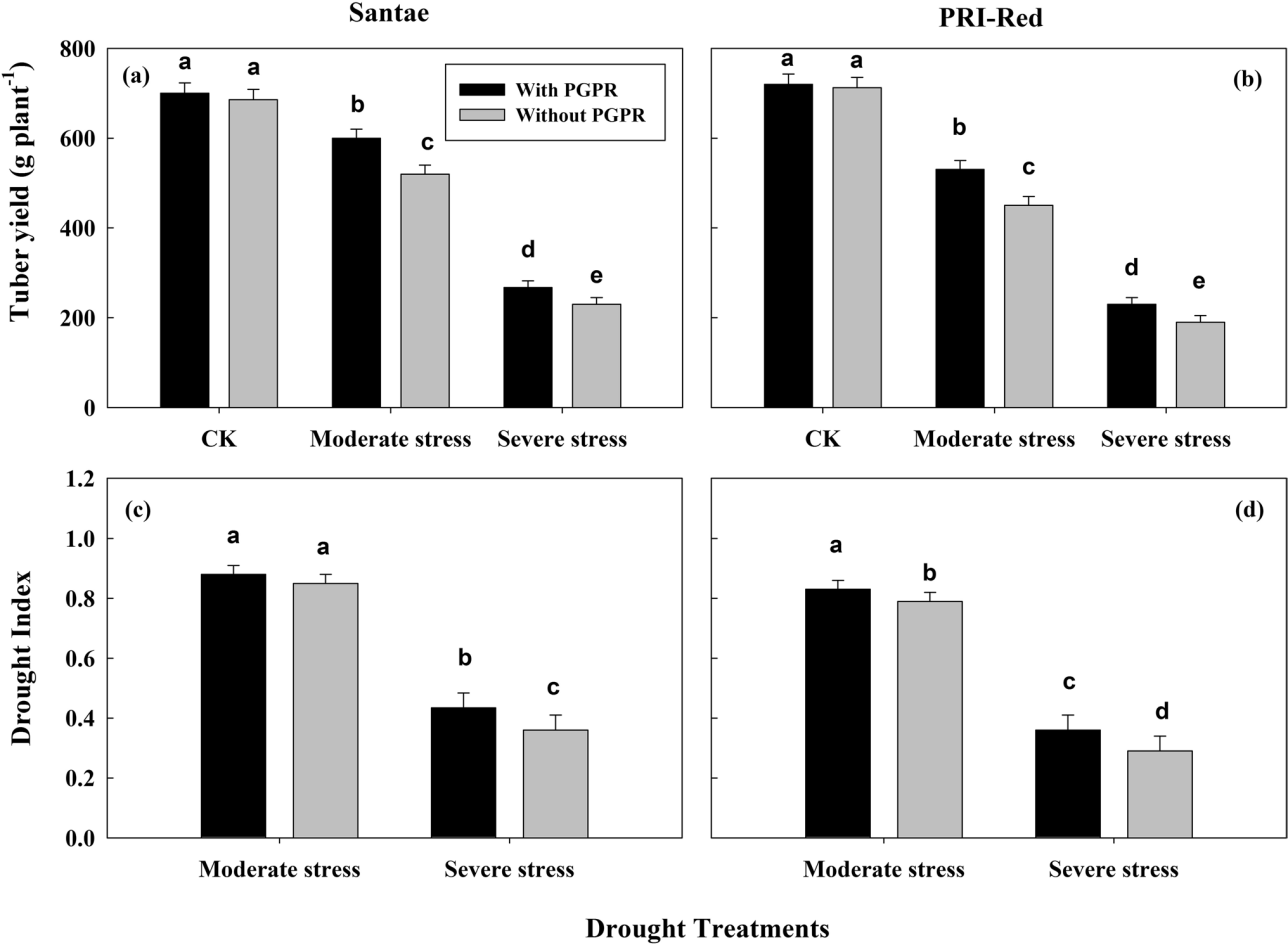
Effect of drought stress and PGPR application on tuber yield (**a**,**b**) and drought index (**c**,**d**) in two potato cultivars. Plants of both genotypes were treated with 100 g of bio-fertilizer at 10 days after germination and exposed to different soil relative water contents (SRWC), including 80 ± 5% (well watered; CK), 60 ± 5% (moderate stress; MS) and 40 ± 5% SRWC (severe stress; SS) for 7 days at tuber initiation stage (30 days after germination). The data is mean of 5 replications. Lowercase letters above bars indicate significant difference between the treatments at P < 0.05.

The drought stresses resulted in a significant reduction in tuber yield per plant in both cultivars (Fig. 1a). However, the plants with PGPR treatments showed higher tuber yield per plant as compared to plants without PGPR under normal and stressed plant conditions. The plants of Santae both with and without PGPR sustained higher tuber yield per plant than the plants of PRI-Red. The tuber yield per plant decreased by 13 and 61% in plants with PGPR and by 25 and 70% in plants without PGPR in Santae; whereas, the plants of PRI-Red with PGPR showed a decrease in tuber yield per plant by 16 and 65%, while without PGPR the decrease in tuber yield was up to 21 and 71% under moderate and severe drought stress as compared to well watered conditions, respectively.

The drought index values decreased as the drought stress increased in both cultivars (Fig. 1b). However, plants with PGPR treatments showed higher drought index values as compared to plants without PGPR. The plants of Santae both with and without PGPR showed higher drought index than the plants of PRI-Red. The values of drought index were as 0.88 and 0.43 in plants with PGPR and as 0.85 and 0.36 in plants without PGPR in Santae; whereas, the plants of PRI-Red with PGPR showed drought index values as 0.83 and 0.36, while without PGPR these values were 0.79 and 0.29 under moderate and severe drought stresses, respectively.

### Changes in leaf relative water content and membrane stability index

**Figure 2:**
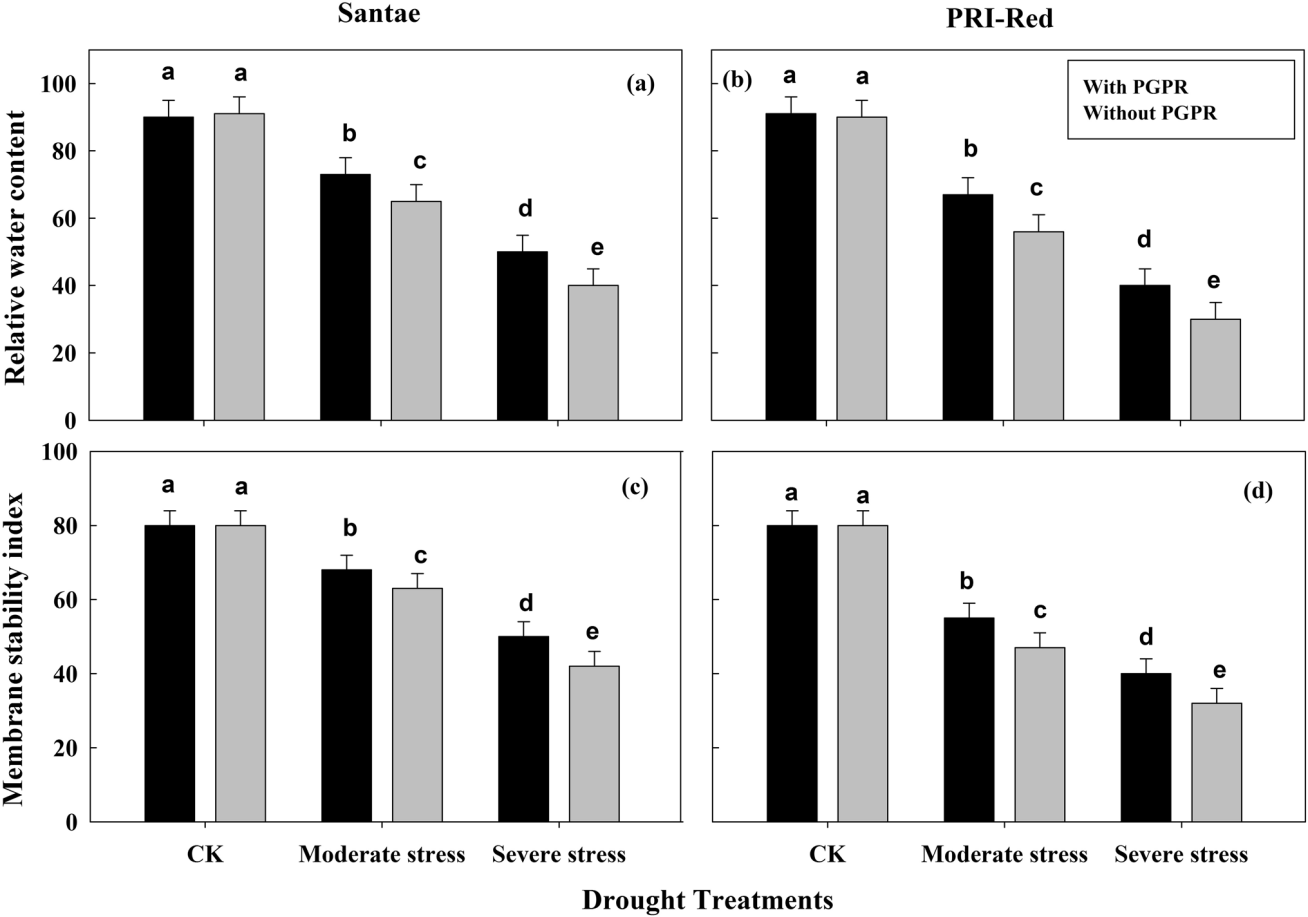
Effect of drought stress and PGPR application on leaf relative water content (**a**,**b**) and membrane stability index (**c**,**d**) in two potato cultivars. Plants of both genotypes were treated with 100 g of bio-fertilizer at 10 days after germination and exposed to different soil relative water contents (SRWC), including 80 ± 5% (well watered; CK), 60 ± 5% (moderate stress; MS) and 40 ± 5% SRWC (severe stress; SS) for 7 days at tuber initiation stage (30 days after germination). The data is mean of 5 replications. Lowercase letters above bars indicate significant difference between the treatments at P < 0.05.

The drought stress decreased relative water content and membrane stability of the leaf in plants of both cultivars (Fig. 2). Under well watered conditions, leaf relative water content and membrane stability were not significantly different in both cultivars. The plants of Santae showed less decrease in leaf relative water content than the plants of PRI-Red (Fig. 2a,b). In drought stressed treatments, the plants with PGPR application showed less decrease in leaf relative water content due to drought stress than the plants without PGPR application. In Santae, leaf relative water content decreased by 15 and 38% in plants with PGPR and by 22 and 47% in plants without PGPR; whereas, the plants of PRI-Red with PGPR showed decrease in leaf relative water content by 29 and 50% and without PGPR the decrease in leaf relative water content was up to 36 and 61% under moderate and severe drought stress, respectively as compared to plants under well watered conditions.

The membrane stability decreased as the level of drought stress increased in both cultivars (Fig. 2c,d). However, in drought stressed treatments, the plants with PGPR showed less decrease in membrane stability index due to drought stress than the plants without PGPR application. Moreover, the plants of Santae showed less decrease in membrane stability index than the plants of PRI-Red. In Santae, membrane stability index was 68 and 51 in plants with PGPR, and 63 and 40 in plants without PGPR; whereas, the plants of PRI-Red with PGPR showed membrane stability index 55 and 40, and without PGPR the membrane stability index was 45 and 32 under moderate and severe drought stress, respectively.

### Changes in chlorophyll and carotenoid contents

**Figure 3:**
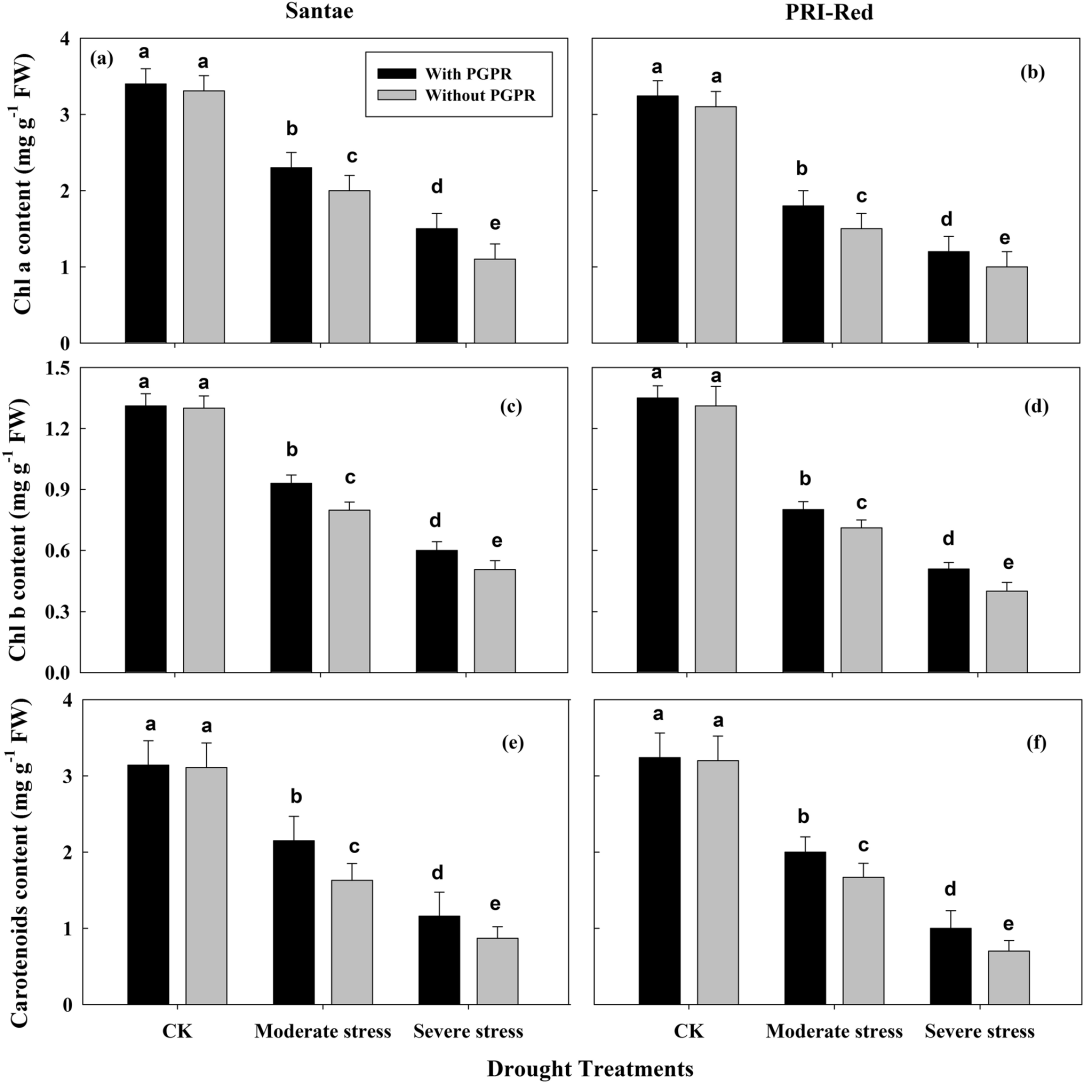
Effect of drought stress and PGPR application on contents of chlorophyll *a* (**a**,**b**), chlorophyll *b* (**c**,**d**) and carotenoids (**e**,**f**) in two potato cultivars. Plants of both genotypes were treated with 100 g of bio-fertilizer at 10 days after germination and exposed to different soil relative water contents (SRWC), including 80 ± 5% (well watered; CK), 60 ± 5% (moderate stress; MS) and 40 ± 5% SRWC (severe stress; SS) for 7 days at tuber initiation stage (30 days after germination). The data is mean of 5 replications. Lowercase letters above bars indicate significant difference between the treatments at P < 0.05.

The drought stress treatments resulted in the reduction of chlorophyll and carotenoid concentrations in the leaves of potato plants of both cultivars (Fig. 3). The plants with PGPR treatments showed higher contents of chlorophyll *a*, *b* and carotenoid as compared to plants without PGPR under drought conditions. The plants of Santae maintained higher chlorophyll *a* content as compared to the plants of PRI-Red (Fig. 3a,b). The chlorophyll *a* content decreased by 28 and 49% in plants with PGPR and by 39 and 67% in plants without PGPR in Santae; whereas, the plants of PRI-Red with PGPR showed a decrease in chlorophyll *a* content by 40 and 61%, while without PGPR the decrease in chlorophyll *a* content was up to 47 and 67% under moderate and severe drought treatments, respectively as compared to well watered conditions.

The drought stress decreased chlorophyll *b* concentration in the leaves of the potato plants in both cultivars (Fig. 3c,d). In drought stressed treatments, the plants with PGPR application showed less decrease in chlorophyll *b* content due to drought stress than the plants without PGPR application. In Santae, chlorophyll *b* content decreased by 27 and 53% in plants with PGPR and by 35 and 63% in plants without PGPR; whereas, the plants of PRI-Red with PGPR showed decrease in chlorophyll *b* content by 33 and 62% and without PGPR the decrease in chlorophyll *b* content was up to 36 and 69% under moderate and severe drought stresses, respectively as compared to plants under well watered treatment.

Similarly, drought stresses resulted in the reduction of carotenoids content in the potato plants of both cultivars (Fig. 3e,f). The plants with PGPR treatments showed less reduction carotenoids content as compared to plants without PGPR both under normal and stressed plant conditions. The plants of Santae contained higher carotenoid contents than the plants of PRI-Red. The carotenoids content decreased up to 28 and 52% in plants with PGPR and to 35 and 63% in plants without PGPR in Santae; whereas, the plants of PRI-Red with PGPR showed reduction in carotenoids content up to 35 and 61%, while without PGPR the reduction in carotenoids content was up to 40 and 71 under moderate and severe drought treatments, respectively as compared to well watered conditions.

### Changes in leaf gas exchange traits

**Figure 4:**
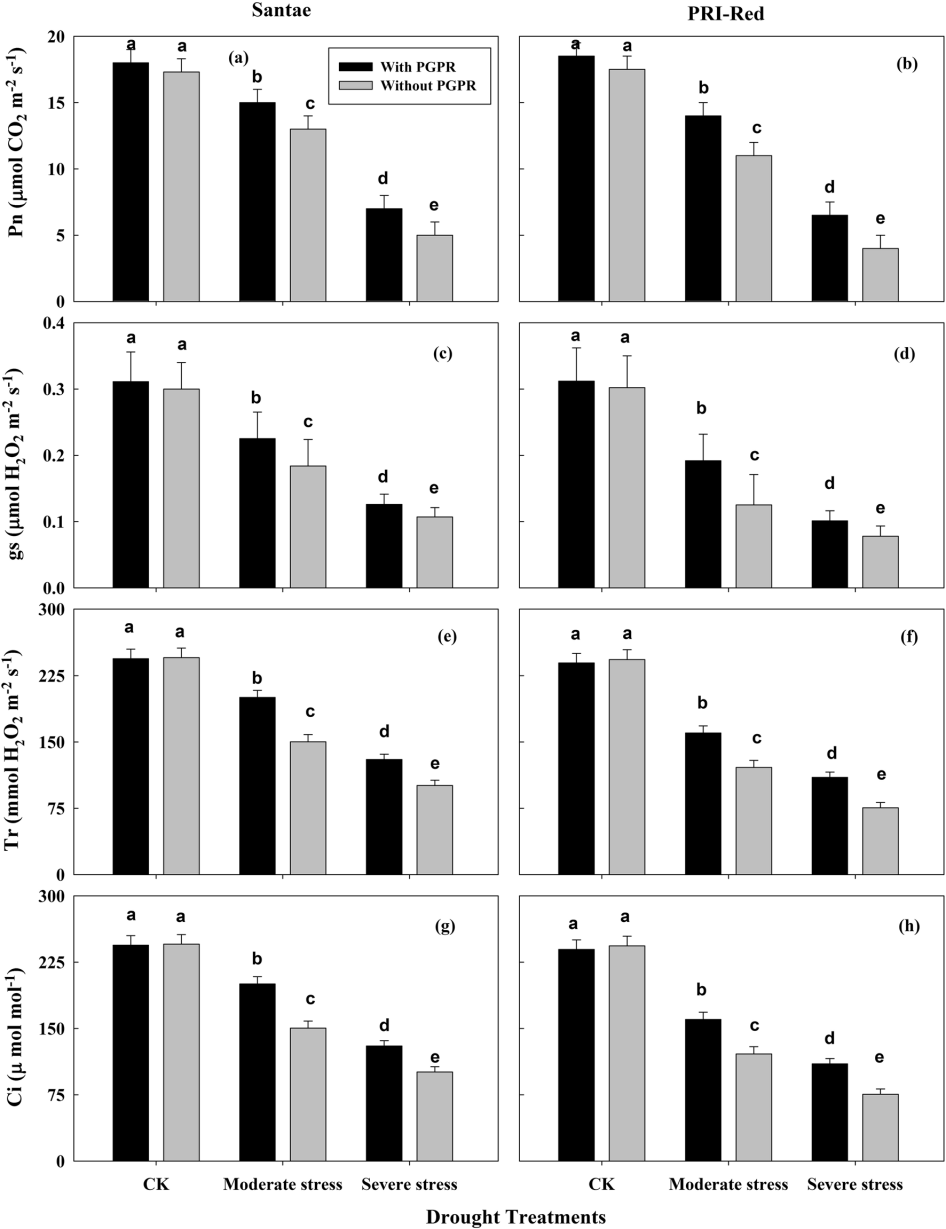
Effect of drought stress and PGPR application on photosynthetic rate (Pn; **a**,**b**), stomatal conductance (gs; **c**,**d**), transpiration rate (Tr; **e**,**f**), and intercellular CO_2_ (Ci; **g**,**h**) in two potato cultivars. Plants of both genotypes were treated with 100 g of bio-fertilizer at 10 days after germination and exposed to different soil relative water contents (SRWC), including 80 ± 5% (well watered; CK), 60 ± 5% (moderate stress; MS) and 40 ± 5% SRWC (severe stress; SS) for 7 days at tuber initiation stage (30 days after germination). The data is mean of 5 replications. Lowercase letters above bars indicate significant difference between the treatments at P < 0.05.

Changes in leaf gas exchange functions, including net photosynthetic rate (Pn), stomatal conductance (Gs), transpiration rate (Tr) and intercellular CO_2_ concentration (Ci) were observed due to drought in the plants of both potato cultivars as shown in Fig. 4.

The drought stresses applied during tuberization stage in the present study resulted in a significant reduction in net photosynthetic rate (Pn) in both cultivars (Fig. 4a,b). Under drought stress, the plants with PGPR displayed less decrease in Pn than plants without PGPR application. For example, during the drought period, Pn decreased by 22 and 58% in plants with PGPR and by 29 and 69% in plants without PGPR in Santae; whereas, the plants of PRI-Red with PGPR showed a decrease in Pn by 30 and 68% and without PGPR the decrease in Pn was up to 35 and 76% under moderate and severe drought treatments, respectively as compared to well watered conditions.

Due to drought stress stomatal conductance (gs) was decreased in both cultivars (Fig. 4c,d). Under well watered conditions, the gs was not significantly different in both cultivars. However, in drought stressed treatments, the plants with PGPR displayed less decrease in gs due to drought stress than plants without PGPR application. In Santae, under moderate and severe drought treatments, gs decreased by 25 and 43% in plants with PGPR and by 32 and 65% in plants without PGPR; whereas under these treatments, the plants of PRI-Red with PGPR showed a decrease in gs by 35 and 63% and without PGPR the decrease in gs was up to 45 and 80%, respectively as compared to well watered conditions.

The drought stress also resulted in a reduction of transpiration rate (Tr) in potato plants of both cultivars (Fig. 4e,f). The plants with PGPR treatments maintained higher Tr as compared to plants without PGPR under stressed plant conditions. The plants of Santae both with and without PGPR maintained higher Tr than the plants of PRI-Red. Tr decreased by 18 and 46% in plants with PGPR and by 23 and 55% in plants without PGPR in Santae; whereas, the plants of PRI-Red with PGPR showed a decrease in Tr by 22 and 51%, while without PGPR the decrease in RGR was up to 28 and 60% under moderate and severe drought treatments, respectively as compared to well watered conditions.

Similarly, drought stresses resulted in a reduction of intercellular CO_2_ concentration (Ci) in potato plants of both cultivars (Fig. 4g,h). The plants with PGPR treatments showed higher Ci as compared to plants without PGPR under drought stress conditions. The plants of Santae both with and without PGPR maintained higher Ci than the plants of PRI-Red. Under moderate and severe drought treatments, Ci decreased by 20 and 46% in plants with PGPR and by 28 and 66% in plants without PGPR in Santae; whereas, the plants of PRI-Red with PGPR showed a decrease in Ci by 30 and 55%, while without PGPR the decrease in RGR was up to 37 and 72% under, respectively as compared to well watered conditions.

### Changes in efficiency and quantum yield of photosystem II

**Figure 5:**
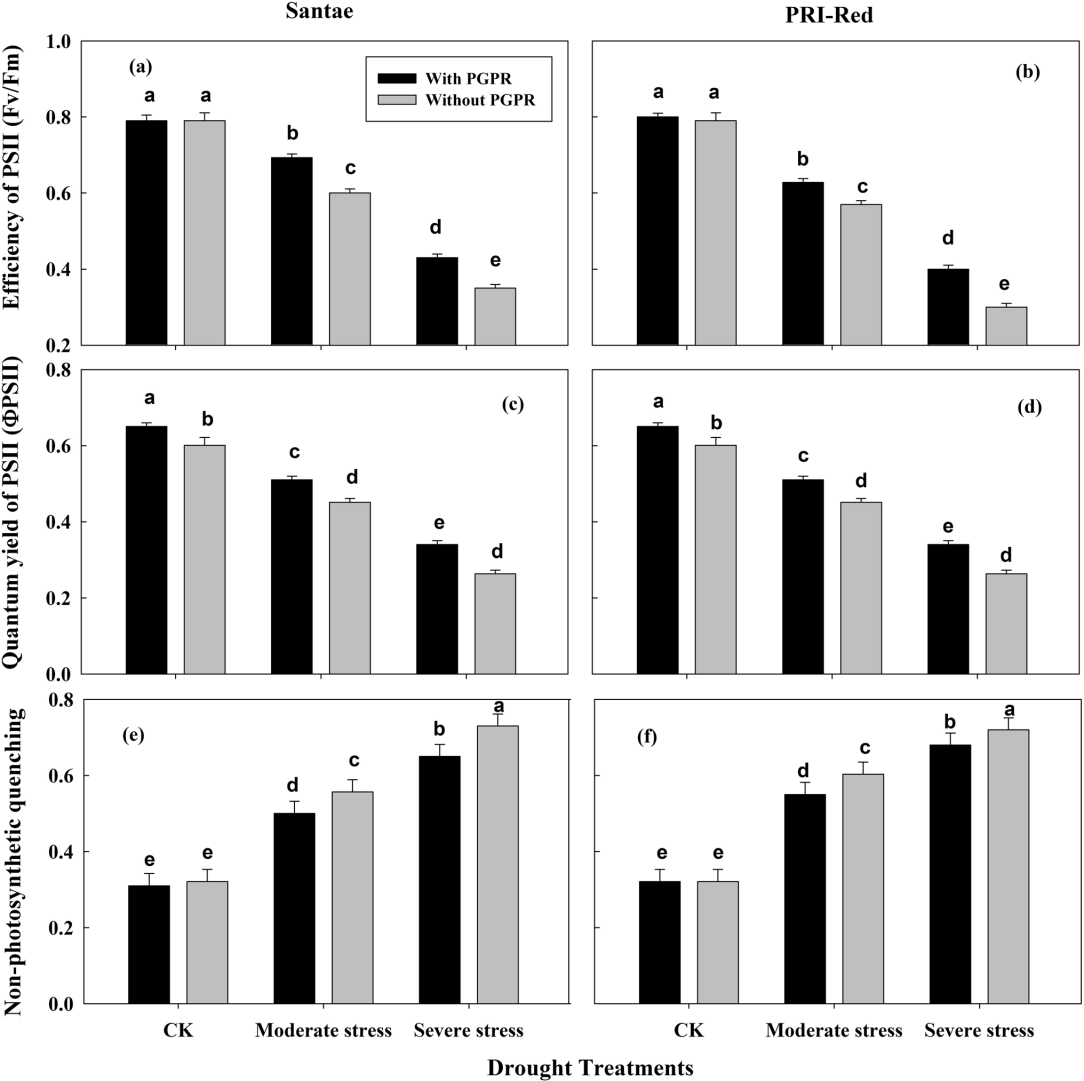
Effect of drought stress and PGPR application on photosynthetic efficiency of PSII (Fv/Fm; Fig. 3a,b), quantum yield of PSII (ΦPSII; **c**,**d**) and non-photosynthetic quenching (NPQ; **e**,**f**) in two potato cultivars. Plants of both genotypes were treated with 100 g of bio-fertilizer at 10 days after germination and exposed to different soil relative water contents (SRWC), including 80 ± 5% (well watered; CK), 60 ± 5% (moderate stress; MS) and 40 ± 5% SRWC (severe stress; SS) for 7 days at tuber initiation stage (30 days after germination). The data is mean of 5 replications. Lowercase letters above bars indicate significant difference between the treatments at P < 0.05.

The drought stress reduced efficiency (Fv/Fm) and quantum yield of photosystem II (ΦPSII) of potato plants of both cultivars (Fig. 5). However, plants with PGPR treatments showed higher Fv/Fm and ΦPSII as compared to plants without PGPR under drought conditions. The plants of Santae maintained greater Fv/Fm and ΦPSII as compared to plants of PRI-Red. For example, under moderate and severe drought treatments, Fv/Fm decreased by 21 and 59% in plants with PGPR and by 30 and 77% in plants without PGPR in Santae; whereas, under these treatments, the plants of PRI-Red with PGPR showed a decrease in Fv/Fm by 30 and 63%, while without PGPR the decrease in Fv/Fm was up to 37 and 87%, respectively as compared to well watered conditions (Fig. 5a,b).

The drought stress decreased ΦPSII of the potato plants in both cultivars (Fig. 5c,d). In Santae, ΦPSII decreased by 22 and 51% in plants with PGPR and by 32 and 68% in plants without PGPR; whereas, the plants of PRI-Red with PGPR showed decrease in ΦPSII by 33 and 60% and without PGPR the decrease in ΦPSII was up to 35 and 69% under moderate and severe drought stresses, respectively as compared to plants under well watered treatment.

In response to decrease in efficiency of PSII, an increase in the non-photosynthetic quenching (NPQ) was observed due to drought stress (Fig. 5e,f). The plants with PGPR application showed less NPQ values as compared to plants without PGPR under stressed plant conditions. The NPQ increased up to 28 and 52% in plants with PGPR and to 35 and 65% in plants without PGPR in Santae; whereas, the plants of PRI-Red with PGPR showed increase in NPQ up to 36 and 63%, while without PGPR the increase in NPQ was up to 41 and 72 under moderate and severe drought treatments, respectively as compared to well watered conditions.

### Changes in reactive oxygen species and lipid peroxidation

**Figure 6:**
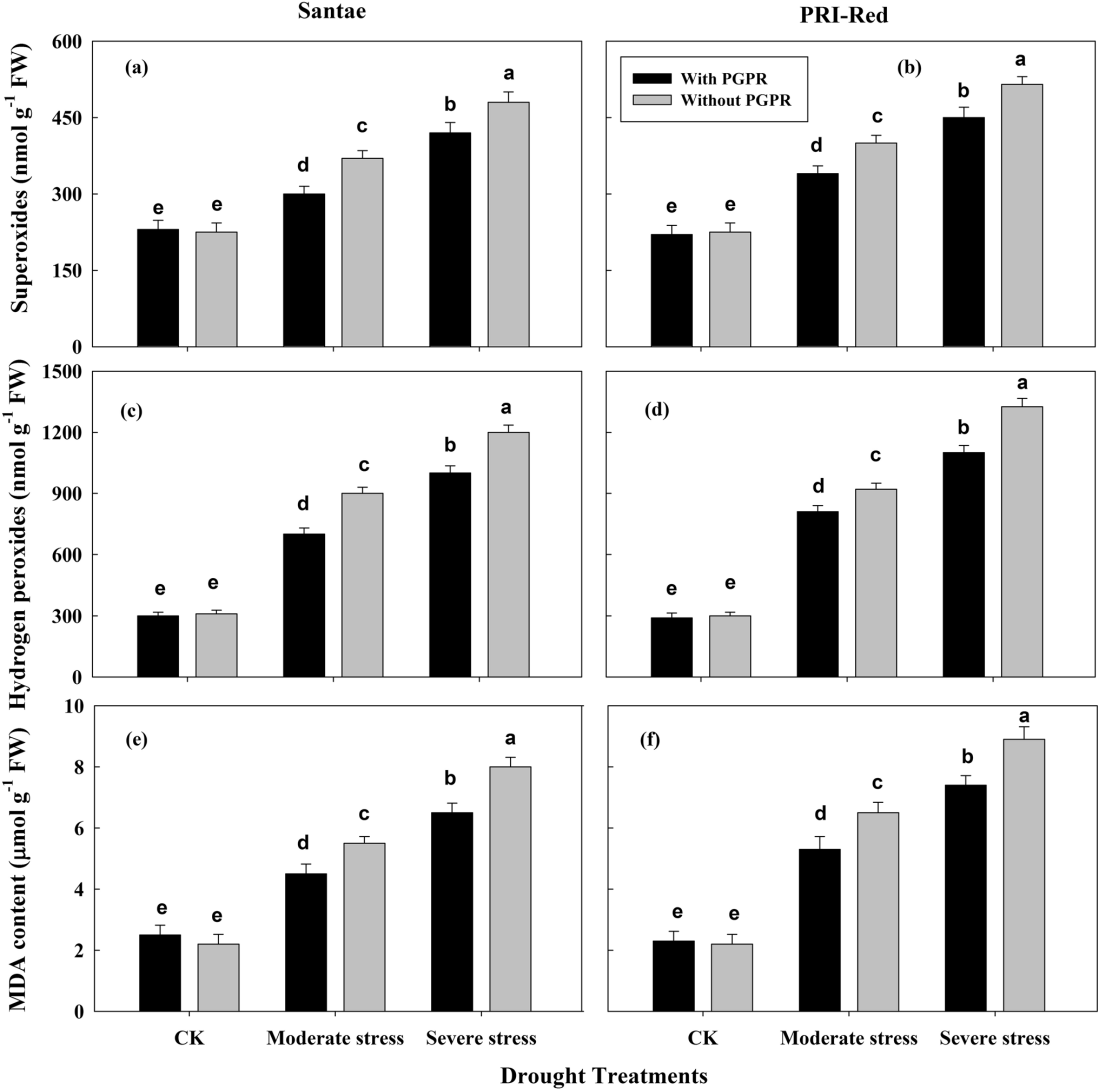
Effect of drought stress and PGPR application on the production of superoxides (**a**,**b**), hydrogen peroxide (**c**,**d**) and malondialdehyde (**e**,**f**) in two potato cultivars. Plants of both genotypes were treated with 100 g of bio-fertilizer at 10 days after germination and exposed to different soil relative water contents (SRWC), including 80 ± 5% (well watered; CK), 60 ± 5% (moderate stress; MS) and 40 ± 5% SRWC (severe stress; SS) for 7 days at tuber initiation stage (30 days after germination). The data is mean of 5 replications. Lowercase letters above bars indicate significant difference between the treatments at P < 0.05.

Changes in reactive oxygen species (ROS), including superoxides and hydrogen peroxides, and malondialdehyde (MDA) were determined due to drought in the plants of both potato cultivars as shown in Fig. 6.

The drought stresses resulted in an increase in the production of superoxides (O_2_^·−^) in both cultivars (Fig. 6a,b). Under drought stress, the plants with PGPR displayed less increase in O_2_^·−^ than plants without PGPR application. During drought, O_2_^·−^ increased by 24 and 48% in plants with PGPR and by 33 and 59% in plants without PGPR in Santae; whereas, the plants of PRI-Red with PGPR showed an increase in O_2_^·−^ by 30 and 58% and without PGPR the increase in O_2_^·−^ was up to 37 and 68% under moderate and severe drought treatments, respectively as compared to well watered conditions.

Due to drought stress the production of hydrogen peroxide (H_2_O_2_) was decreased in both cultivars (Fig. 6c,d). However, under drought stress treatments, the plants with PGPR displayed less increase in H_2_O_2_ than plants without PGPR application. In Santae, under moderate and severe drought treatments, H_2_O_2_ increased by 47 and 68% in plants with PGPR and by 56 and 75% in plants without PGPR; whereas under these treatments, the plants of PRI-Red with PGPR showed an increase in H_2_O_2_ by 55 and 73% and without PGPR the decrease in H_2_O_2_ was up to 62 and 85%, respectively as compared to well watered conditions.

The drought stress resulted in an increase in lipid peroxidation measured by production of malondialdehyde (MDA) (Fig. 6e,f). However, plants with PGPR treatments showed less MDA production as compared to plants without PGPR under drought stress. The plants of Santae both with and without PGPR showed less MDA production than the plants of PRI-Red. MDA content increased by 41 and 65% in plants with PGPR and by 50 and 75% in plants without PGPR in Santae; whereas, the plants of PRI-Red with PGPR showed an increase in MDA content by 52 and 75%, while without PGPR the increase in MDA production was up to 58 and 79% under moderate and severe drought treatments, respectively as compared to well watered conditions.

### Changes in activities of enzymatic antioxidants

**Figure 7:**
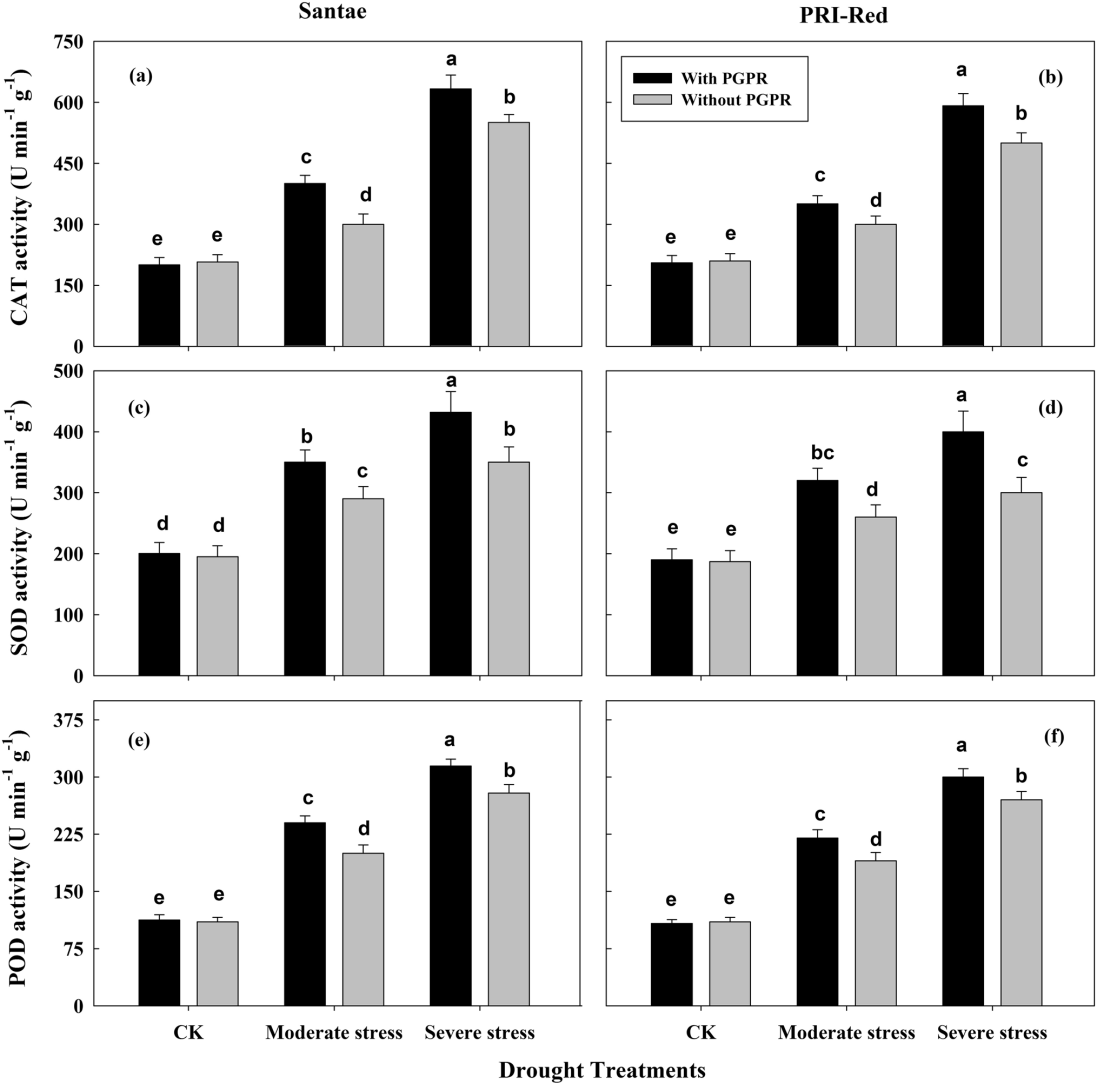
Effect of drought stress and PGPR application on enzymatic activities of catalase (CAT; **a**,**b**), superoxide dismutase (SOD; **c**,**d**) and peroxidase (POD; **e**,**f**) in two potato cultivars. Plants of both genotypes were treated with 100 g of bio-fertilizer at 10 days after germination and exposed to different soil relative water contents (SRWC), including 80 ± 5% (well watered; CK), 60 ± 5% (moderate stress; MS) and 40 ± 5% SRWC (severe stress; SS) for 7 days at tuber initiation stage (30 days after germination). The data is mean of 5 replications. Lowercase letters above bars indicate significant difference between the treatments at P < 0.05.

The activity of catalase (CAT) increased due to drought stresses in potato plants of both cultivars (Fig. 7a,b). It was higher under severe drought stress than moderate stress. Under drought stress, the plants with PGPR treatments showed higher CAT activity as compared to the plants without PGPR application. CAT activity increased up to 37 and 68% in plants with PGPR and to 31 and 54% in plants without PGPR in Santae; whereas, the plants of PRI-Red with PGPR showed increase in CAT activity up to 30 and 61%, while without PGPR, the CAT activity increased up to 28 and 56% under moderate and severe drought treatments, respectively as compared to well watered conditions.

Under drought stress, the activity of superoxide dismutase (SOD) increased (Fig. 7c,d). It was higher under severe stress as compared to moderate drought stress. Under drought stress, the plants with PGPR treatments showed higher SOD activity as compared to the plants without PGPR application. SOD activity increased up to 24 and 63% in plants with PGPR and to 20 and 50% in plants without PGPR in Santae; whereas, the plants of PRI-Red with PGPR showed increase in SOD activity up to 20 and 54%, while without PGPR the SOD activity increased up to 14 and 46% under moderate and severe drought treatments, respectively as compared to well watered conditions.

Similarly, the activity of peroxidase (POD) increased (Fig. 7e,f). It was the maximum under severe stress but decreased at moderate drought stress. The plants with PGPR showed higher POD activity as compared to the plants without PGPR application under drought stress. SOD activity increased up to 39 and 66% in plants with PGPR and by 30 and 56% in plants without PGPR in Santae; whereas, the plants of PRI-Red with PGPR showed increase in POD activity by 27 and 58%, while without PGPR the POD activity increased by 22 and 51% under moderate and severe drought treatments, respectively as compared to well watered conditions.

### Changes in production of osmolytes

**Figure 8:**
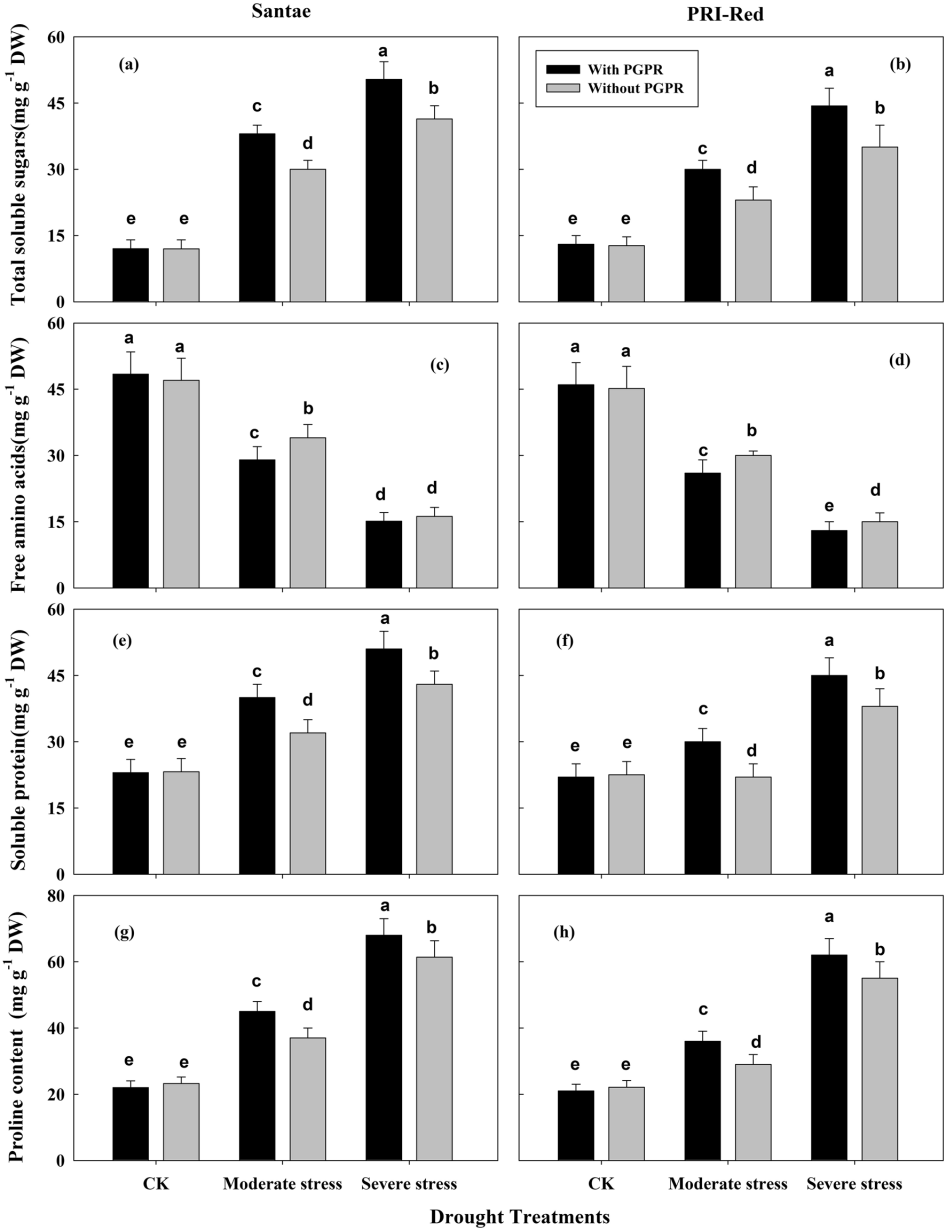
Effect of drought stress and PGPR application on total soluble sugars (Fig. 4a,b), free amino acids (Fig. 4c,d), soluble proteins (Fig. 4e,f), and proline contents (Fig. 4g,h) in two potato cultivars. Plants of both genotypes were treated with 100 g of bio-fertilizer at 10 days after germination and exposed to different soil relative water contents (SRWC), including 80 ± 5% (well watered; CK), 60 ± 5% (moderate stress; MS) and 40 ± 5% SRWC (severe stress; SS) for 7 days at tuber initiation stage (30 days after germination). The data is mean of 5 replications. Lowercase letters above bars indicate significant difference between the treatments at P < 0.05.

Changes in the production of osmolytes, including total soluble sugars, free amino acids, soluble protein, and proline contents were recorded in the plants of both potato cultivars as shown in Fig. 8.

The production of total soluble sugars increased due to drought stresses in potato plants of both cultivars (Fig. 8a,b). Its production increased as the level of drought stress increased. Under drought stress, plants with PGPR treatments showed higher production of total soluble sugars as compared to the plants without PGPR application. The production of total soluble sugars increased by 59 and 75% in plants with PGPR and by 51 and 67% in plants without PGPR in Santae; whereas, the plants of PRI-Red with PGPR showed increase in production of soluble sugars up to 42 and 71%, while without PGPR the total soluble sugars increased up to 36 and 66% under moderate and severe drought treatments as compared to well watered conditions, respectively.

The contents of free amino acids decreased due to drought stresses in potato plants (Fig. 8c,d), which decreased as the drought stress increased. Under drought stress, the plants with PGPR treatments showed lower free amino acids as compared to the plants without PGPR. The contents of free amino acids decreased by 32 and 55% in plants with PGPR and by 24 and 60% in plants without PGPR in Santae; whereas, the plants of PRI-Red with PGPR showed decrease in free amino acids up to 20 and 42%, while without PGPR the free amino acids decreased up to 21 and 67% under moderate and severe drought treatments as compared to well watered conditions, respectively.

Due to drought stresses the contents of soluble proteins increased in potato plants of both cultivars (Fig. 8e,f). It increased as drought level increased. More production of protein was observed in plants treated with PGPR than plants without PGPR. The contents of soluble proteins increased up to 25 and 68% in plants with PGPR and by 20 and 59% in plants without PGPR in Santae; whereas, the plants of PRI-Red with PGPR showed an increase in production of soluble proteins up to 20 55%, while without PGPR the contents of soluble proteins increased up to 17 and 46% moderate and severe drought treatments as compared to well watered conditions, respectively.

Similarly, drought stresses resulted in an increase in production of proline (Fig. 8g,h). Under drought stress, the plants with PGPR treatments showed higher production of proline as compared to the plants without PGPR application. The production of proline increased by 41 and 69% in plants with PGPR and to 34 and 60% in plants without PGPR in Santae; whereas, the plants of PRI-Red with PGPR showed an increase in production of proline up to 25 and 57%, while without PGPR the proline content increased up to 21 and 50% under moderate and severe drought treatments as compared to well watered conditions, respectively.

## Discussion

Drought, the most important abiotic stress in agriculture, is of particular importance in potato (*Solanum tuberosum* L.) due to the susceptibility of this crop to water deficit which results in considerably reduced tuber number and weight as well as in the loss of tuber quality^10,28^. The current potato cultivars are highly yielding but very susceptible to drought stress^31^. The drought occurrence during the tuber initiation stage causes substantial decrease in tuber yield associated with disturbed plant physiological and biochemical activities. Research on drought tolerance in potato is still limited but in the last few years has increased in response to the expansion of its cultivation to drought prone areas^32^. Identifying water stress tolerance traits in potato has received increased interest, principally for their possible use as indirect selection criteria in breeding programs^33^.

The use of beneficial microbes such as plant growth- promoting rhizobacteria (PGPR) in agricultural production systems started a long time ago, and there is increasing evidence that beneficial microbes can enhance plants’ tolerance to adverse environmental stresses^34,35^. However, physiological significance of PGPR in terms of photosynthetic activity and its effects on final tuber yield under drought stress are not yet explained in potato crop. In this study, it was observed that application of PGPR reduced the impact of drought on growth and physio-chemical traits of the plants and enhanced the ability of potato plants to maintain favorable physiological, biochemical and agronomic traits under drought stress. We studied a wide range of plant strategies and role of PGPR to cope with drought stress, and discussed how regulation of leaf plant functions respond to severe and moderate drought stress under control conditions. These study conditions made it possible to measure extensive traits during the experiment those can be exploited to field level with some confidence.

The drought treatments applied during tuberization stage resulted in a significant reduction of relative growth rate (RGR) and plant dry matter production in both cultivars (Table 1). Severe drought stress affected the crop establishment, growth and final productivity through its strong interference with plants physiological processes. A greater decline in RGR in plants under severe stress might be associated with higher degree of photosynthetic decline during drought stress and lower degree of photosynthetic recovery after re-watering. When drought-stressed plants were rewatered, the RGR was almost recovered to the level of plants at 80% soil relative water contents (SRWC), which might suggest a reversible trend of the physiological fluctuations from the downregulated values caused under drought stress^14^. Despite the RGR restored after re-watering, the total dry matter production measured on maturity could not reach the level of the well watered treatment in both cultivars indicating the existence of pre-drought limitations. The plants with PGPR maintained greater RGR and dry matter production and consequently showed less pre-drought limitation values. Growth promoting effects of PGPR have also been reported in previous studies^7,9,36^.

Some of the responses appeared in leaf traits in response to changes occurred in the leaf itself or elsewhere in the whole plant ultimately determined the adaptability of potato cultivars (Table 2). Under drought stress, the cultivar PRI-Red showed smaller leaf area with higher leaf dry matter content as compared to Santae, which might be a reflection of higher density of chloroplasts and thus higher chlorophyll per unit leaf area. Such a leaf adaptation might enable the plant to gain more carbon over unit water consumption, which might result in better light interception and higher water use efficiency. Such adaptations might also enhance the plant capability to overcome the photoinhibition conditions due to drought stress^19^. Under water limitation conditions, leaf area reduction characteristics likely had saved soil water reserves for the next stages of plant development through minimized transpirational losses. It was observed that reduced leaf area with improved leaf dry matter content played a key role in leaf and plant functioning, which is a key genotype-associated tactic of water acquisition and its efficient utilization^37^.

A variation in final tuber yield of plants with PGPR and without PGPR was observed due to the differences in tuber number and their weights (Fig. 1). The plants under PGPR treatment showed an ability to sustain a higher number of tubers and weights. Moreover, an extended period of leaf photosynthetic capacity during the phase of tuber formation was observed in PGPR treated plants which might have contributed to the production of sufficient amounts of photoassimilates and tubers with higher weights. Our findings of extended period of photosynthetic capacity during tuber formation phase correlated with the production of higher tuber yield were in accordance with the findings of^16^ and^30^. Analysis of variance indicated that cultivars, PGPR and drought stress all had a highly significant (P < 0.05) effect on crop growth, tuber yield and drought indexes. Santae out-yielded PRI-Red under drought stress conditions as compared to well watered conditions. Similar findings showing the effects of drought on number of tubers per plant and tuber weights have also been reported by earlier researches^5,22,26^. However, previous drought stress experiments are normally based on days after sowing or emergence without a proper consideration of the phenological stage of potato crop, a factor that must be considered when the aim is to compare the physiological responses of dissimilar genotypes to drought occurring at similar growth stages.

Plant growth and production are the outcomes of photosynthesis and key interlinked-physiological processes, functioning in the leaf^2^. Photosynthesis, stomatal conductance, intercellular CO_2_ concentration and transpiration occur in parallel with each other under drought stress conditions, such that decrease in the efficiency of one will result in the downregulation of the others^23^. In our study, under drought stress, plant showed decline in photosynthesis, stomatal conductance, intercellular CO_2_ concentration, and transpiration rate (Fig. 4a,b). At relatively moderate water deficit, stomata of potato leaf are closed which result in reduced photosynthesis^27^ and limited photosynthesis under abiotic stresses becomes the main cause of losses in biomass and yield of the plant^6^. Therefore, an inhibited growth in drought stressed plants might be associated to the decreased rate of photosynthesis, which might have resulted in limited cell growth and development. However, plants under PGPR treatment showed an ability to maintain a higher stomatal conductance, photosynthetic rate, intercellular CO_2_ concentration and transpiration in their leaves as compared to the plants without PGPR applications, indicating a sustained plant health and growth.

A major consequence of drought stress was reflected by induced chlorophyll degradation in the leaves (Fig. 2). A decline in chlorophyll contents during drought stress accelerated a reduction in the photochemical activities of chloroplasts, which might be responsible for the decrease in photosynthesis. Under drought stress, damage to the chloroplasts might lead to photo-inhibition, which decreases the photosynthetic energy (ATP) consumption in the Calvin Cycle due to decreased electron transport rate^38^. Sufficient chlorophyll availability might improve photosynthetic capacity or stomatal control under water deficit conditions, because more than half of the crop green area is active in collecting light to drive the photosynthesis^13^. Hence, the rate of photosynthesis per unit leaf area can be enhanced by increasing the total amount of photosynthetic pigment (chlorophyll) per unit leaf area, for which application of an optimal amount of PGPR may be a key strategy. Moreover, the maintenance of plant’s water status in the form of leaf relative water contents and stomatal opening possibly associated with PGPR were important to maintain the stomatal conductance for CO_2_, the running of photosynthetic reactions and working of electron transport chain.

The extent of reactive oxygen species (ROS) and malondialdehyde (MDA) accumulation in leaves depended on the severity of drought stress and the PGPR (Fig. 6). The intercellular concentration of MDA indicates the extent of oxidative stress^1^. MDA damage might trigger the multiple developmental signals characterized by the loss of chlorophyll, increased cell membrane permeability, the breakdown of macromolecules, massive remobilization of nutrients and early senescence, ultimately reducing the period for crop development. The higher ROS and MDA contents observed in plants without PGPR during drought stress (Fig. 6) indicate a suppressed capacity of sub-cellular antioxidant system and higher accumulation of reactive oxygen species in these plants. It is considered that the inhibition of photosynthesis process also leads to the accumulation of reactive oxygen species in the cell organelles^3,15^. A tight control of ROS generation and accumulation in the plant cells is essential; otherwise, over-produced ROS can cause cell death by reducing the plant growth and finally the economic yield loss^8,39^. The lower MDA and ROS contents in plants with PGPR as compared to without PGPR plants indicate an improved reactive oxygen species scavenging capability under drought conditions.

The higher enzymatic antioxidant activities of CAT, SOD and POD with lower MDA contents identified in plants with PGPR indicated their improved redox defense status to scavenge reactive oxygen species damage by down-regulating peroxidation of cell membrane lipids as well as structural and functional proteins under drought stress (Fig. 7). Our results indicated that development of favorable ROS detoxifying antioxidant system in the drought stressed plants under PGPR might have contributed towards the protection of the photosynthetic process. Similar results were reported by^40^, who suggested that PGPR nutrition contributed to the drought tolerance in *Agrostis palustris* (bent grass) by preventing the cell membrane damage, lower MDA accumulation and higher osmoregulation.

Similarly, plants can adjust water relations to maintain cellular functions under drought conditions^19^. Plants develop osmotic adjustment by synthesizing and accumulating compatible solutes such as soluble sugars, protein, free amino acids, and proline^40^. The accumulation of compatible solutes allows the plant to maintain turgor pressure and cell volume at low water potential, which is important for maintaining metabolic functions^39,41,42^. Moreover, accumulation of osmolytes facilitates the recovery of metabolic activities after relief from stress^43^.

## Conclusions

Plant growth, yield and physio-chemical functions of potato plants were largely influenced by drought stress. The decrease in the plant physiological, biochemical and agronomic characteristics increased as the level of drought stress increased. However, the PGPR treatment benefited the plants to maintain their development and functions efficiently, ultimately providing the growth promotion. The plants with PGPR sustained higher dry matter production, leaf area, tuber number and weight as compared to the plants without PGPR. The plants under PGPR treatment showed lower decrease in membrane stability, leaf relative water contents, chlorophyll and carotenoid contents, photosynthetic functions, and finally in tuber yield of plants. These plants showed less ROS and MDA production and maintained higher enzymatic activity of CAT, SOD and POD with increased production of total soluble sugars, total soluble proteins and proline. The present study gives new insights about various effects of PGPR on morphological, physiological and biochemical traits in response to drought stress in potato. Such information can further provide practical guidelines towards the research efforts for improving drought tolerance and yield sustainability in potato crops.

## Material and methods

To investigate the effect of plant growth regulators rhizobacteria (PGPR) on different potato genotypes under drought stress, a pot experiment was conducted from October to February, 2017–2018. Detail about the materials which were used and the techniques adopted during the experimentation, plant sampling and analysis are as described here.

### Experimental site

Pot experiment was conducted in the Department of Botany at University of Sargodha (32.1° N, 72.67° E), Pakistan. The laboratory studies were carried out at Plant Tissue Culture Laboratory, Department of Botany, University of Sargodha.

### Germplasm detail

Seeds of two potato genotypes, namely Santae and PRI-Red were obtained from Punjab Seed Corporation, Sahiwal, Pakistan. These cultivars are widely cultivated in the middle and lower Indus River Basin and show almost similar phenology and yield potentials under optimum field conditions. Pre-experiments were performed to monitor the behavior of cultivars to the drought stress. The cultivar Santae was classified as drought-tolerant while PRI-Red as drought-sensitive. Santae is Holland originated white colour cultivar, while PRI-Red is red in colour and developed by Punjab Potato Research Institute.

### Experimental design

The experiment was conducted under a rain exclusion shelter of polythene plastic sheet. The experiment was laid out in a Randomized Complete Block Design (RCBD) with the factorial arrangement. Two potato cultivars, three water levels and two PGPR applications (with and without) were arranged as first, second and third independent factor, respectively. Each treatment had 20 replicates (pots). The pots with different treatments were arranged in RCBD layout.

### Plant culture and growth conditions

Before sowing, seeds of both cultivars were surface-sterilized by soaking in 1% (w/v) sodium hypochlorite solution for 20 min and rinsed three times with sterile deionized water. Uniform selected seeds were planted in free-draining plastic pots with 30 cm height and 25 cm diameter. One seed per pot was planted and each pot was filled with 7 kg air-dried, sieved and uniformly mixed sandy-clay loam soil having 15% soil moisture. At the time of soil filling, 1.0 g N, 0.5 g P_2_O_5_ and 1.1 g K_2_O per pot were applied for each treatment. Further, 0.5 g per pot N was applied at 20 days after sowing and at tuberization stage, respectively. Each pot was irrigated at 80% soil relative water content (SRWC) with tap water characterized with 7.5 pH, 2.8 dsm^−1^ electrical conductivity and 1200 mg L^−1^ total soluble salts until the start of water deficit treatments.

### Treatments application

#### Plant growth regulators rhizobacteria application

Isolates of bacterial strains (*Bacillus* sp.) were obtained from Ayub Agricultural Research Center, Faisalabad, Pakistan. *Bacillus subtilis* HAS31 (16S rRNA gene partial sequence accession = MT658521; https://www.ncbi.nlm.nih.gov/nuccore/MT658521) was used as PGPR strain. To prepare 100 g inoculant of HAS31, 45 g of peat was mixed thoroughly with 5 g of CaCO_3_, poured 20 mL of water (pH 6.8) and stored in sealed polythene bags. The peat filled polyethylene bags were sterilized in autoclave. HAS31 was cultured on Minimal salt medium and purified overnight 30 mL liquid bacterial culture was added aseptically to each sterilized bag and incubated for 5 days at 37 ± 2 °C. After every 48 h the peat was mixed by shaking the inoculated bags. After 10 days of germination, the 100 g bags of inoculants were applied to the half pots under each treatment.

#### Drought stress application

The experiment was designed including three water levels for both cultivars. The pots were irrigated with tap water daily at field capacity till the onset of drought application stage (tuber initiation stage, 30 days after germination). For drought stress application, irrigation to pots was withheld until soil relative water content (SRWC) reached at 60 ± 5% and 40 ± 5% for moderate and severe drought stress, respectively. These water deficits were maintained for 7 days by daily estimating soil moisture contents in the pots and compensating the water lost. Meanwhile, the control pots were kept well-watered at 80 ± 5% SRWC. After the water deficits application for 7 days, all pots were re-watered to well-watered level until maturity of the crop. Soil water status was estimated before water application to pots by using the following equation:

1 $$ {\text{SRWC}}\; (\% ) = \frac{{{\text{FW}} - {\text{DW}}}}{{{\text{TW}} - {\text{DW}} }} \times 100 $$

where, FW is fresh weight, DW is the dry weight and TW is the saturated soil weight estimated by saturating soil samples for 24 h and calculating the amount of water as given by Rhoades, 1996^44^.

#### Plant sampling

On the last day of drought stress, plant samples were collected for the measurements of plant water status, chlorophyll contents, lipid peroxidation and antioxidative activities of superoxide dismutase (SOD) and catalase (CAT), PPO, APL, total soluble sugars, soluble proteins, free amino acids, proline, fresh and dry biomass of plants.

#### Harvesting

The plants were harvested at the end of January, 2018 and the agronomic measurements like plant height, number of tubers, number of leaves, tuber weight/yield per pot, leaf area and leaf area duration were estimated.

### Traits measurements

#### Measurements of morphological traits

Leaf area (LA) per plant was recorded in cm^2^ with the help of leaf area meter. After measuring LA, the leaves were rehydrated overnight at 4 °C to get the turgid weight (TW). Then leaves were oven-dried at 70 °C for 48 h to get the dry weight (DW). After this, specific leaf area (SLA = LA/DW) and leaf dry matter contents (LDMC = DW/FW) were calculated. Plant height was recorded in cm with the help of meter tap.

At maturity, the whole plant in the pot was cut with a sharp scissor from the ground level. To get dry mass of the plants, the samples were oven dried at 70 °C for about 80 h till the constant weights were achieved. At maturity, number of leaves per plant was calculated by counting all the leaves in a plant. Height of plants under different treatments was recorded in cm by using meter tap. All potatoes in a pot were collected and counted to record number of tubers per plant. Tuber yield per plant was calculated by weighing all the potatoes per plants under different treatments.

#### Pre-drought limitation

Pre-drought limitation (PDL) in the production of dry matter due to drought treatments was estimated at maturity by the following equation:

2 $$ {\text{PDL}}\; (\% ) = \frac{{{\text{DMC }}{-}{\text{ DMT}}}}{{\text{ DMC }}} \times 100 $$

where, DMC stands for dry matter under control; whereas, DMT defines dry matter under treatments.

#### Drought index

Drought index (DI) was estimated by the tuber yield differences between the plant under drought stress treatments and the plants in the control pots according to the method given by Huang and Zhao, 2001^45^;

3 $$ {\text{DI}} = \frac{{{\text{YD}}}}{{\text{ YC }}} $$

where, YD is the grain yield under drought stress treatments and YC is the grain yield under control.

#### Measurements of biochemical characters

Quantitative estimation of biochemical contents, including Polyphenol Oxidase (PPO), phenylalanine ammonium lyase (PAL), total soluble sugars (TTS), free amino acids (AA), soluble proteins, and activities of enzymes like Catalase (CAT), superoxide dismutase (SOD) and peroxidase (POD) were investigated by using UV-1100 absorption spectrophotometer at Hi tech Laboratory of the university.

#### Extraction of plant sample

Under different treatments, 1 g of leaves were weighed with electrical balance and crushed into fine powder with the help of a pestle and mortar using liquid nitrogen. After crushing, 10 mL of 0.02 M phosphate buffer (pH 7.0) was added to the powder and slurry was formed that was transferred to 1.5 mL Eppendorf tubes. To separate the supernatant for the mixture, Eppendorf tubes were centrifuged at 12,000 rpm for 10 min in centrifuge (MIKRO 120 Hettich Zentrifugen 4108). The supernatant was removed by using micropipette and transferred to another Eppendorf tube for biochemical analysis.

#### Total soluble sugar contents

The method given by Yemm and Willis, 1954^46^ was used to determine total sugar contents by using anthron and 80% Sulphuric Acid (H_2_SO_4_). Test tubes with the mixture were placed in water bath for 10 min, heated and then cooled in ice water. The optical density was observed at wavelength of 620 nm.

#### Total soluble proteins

Biuret method as described by Roensen and Johnson, 1961^47^ was used for the estimation of total soluble protein contents. After adding the required chemical constituents and shaking vigorously, the tube were incubated at room temperature for 25 min. To observe the optical density on UV-spectrophotometer, wavelength of 545 nm was used. From bovine serum albumin a standard curve for protein was obtained to calculate the total protein content.

#### Determination of reactive oxygen species, lipid peroxidation and enzymatic antioxidant activities

Reactive oxygen species, lipid peroxidation (estimated by measuring the malondialdehyde contents: MDA) and antioxidant activities in the leaf were determined following the methods given by Tang et al. 2010^48^ and Zhang et al. 2012^49^. Fresh leaf samples (0.5 g) were sliced and homogenized in a mortar and pestle with 5 mL ice-cold extraction buffer containing 50 mM potassium phosphate buffer (pH 7.0) and 0.4% polyvinylpoly pyrrolidone (PVP). The homogenates were centrifuged at 10,000×*g* for 30 min at 4 °C. Then supernatants were collected and used as crude extracts for the above-cited assays by using a Pharmacia Ultra Spec Pro UV/VIS spectrophotometer (Pharmacia, Cambridge, England).

Superoxides (O_2_^·−^) were determined with a reaction mixture of 0.5 mL phosphate buffer (pH 7.8), 1 mL 1 mM hydroxylammonium chloride, 1.0 mL 17 mM P-aminobenzene sulfonic acid and 1.0 mL 7 mM α-naphthylamine. The mixture was incubated at 25 °C for 60 min and absorbance was noted at 530 nm. Hydrogen peroxide (H_2_O_2_) was determined after isolation by peroxidase coupled assay using 4-aminoantipyrine and phenol as donor substrates. The carbonyl content in oxidatively modified proteins was quantified using the 2, 4-dinitrophenylhydrazone assay procedure by recording the absorbance at 290 nm. Contents of MDA were determined through thiobarbituric acid (TBA) method at 532 nm, and then corrected by subtracting non-specific absorbance values at 600 nm by using an extinction coefficient of 156 mmol L^−1^ cm^−1^.

Superoxide dismutase (SOD) activity was determined according to Tang et al. 2010^48^ by adding 0.1 mL enzyme extract to a reaction mixture of 1.5 mL 50 mM sodium phosphate (pH 7.8), 0.3 mL 130 µM methionine, 0.3 mL 750 µM nitro-blue tetrazolium (NBT), 0.3 mL 100 µM EDTA-Na_2_, 0.300 mL 20 µM riboflavin and 100 µL distilled water, and illuminated under light of 4000 flux for 20 min and then sample absorbance was determined at 560 nm. One unit of SOD activity was considered as the amount of enzyme used for 50% inhibition of the NBT reduction. Peroxidase (POD) activity was determined by adding 50 µL enzyme extract to a reaction mixture containing 1.0 mL 50 mM sodium phosphate (pH 5.5), 1.0 mL 0.3% H_2_O_2_ and 0.95 mL of 0.2% guaiacol. The absorbance value at 470 nm for changes in a unit of POD enzyme activity was noted. For catalase (CAT) activity, 200 µL enzyme extract was added to the reaction mixture of 1.5 mL 50 mM sodium phosphate (pH 7.8), 300 µL 0.1 M H_2_O_2_ and 1.0 mL distilled water. The change in absorbance at 240 nm per minute as a unit of CAT activity was recorded. For ascorbate peroxidase (APX) activity, 200 μL enzyme extract was added to a reaction mixture of 50 mmol L^−1^ potassium phosphate buffer (pH 7.0), 0.5 mmol L^−1^ ASC and 0.1 mmol L^−1^ H_2_O_2_. APX activity was determined by noting the decrease at 290 nm for 1 min in 1 mL of the reaction mixture.

### Measurements of physiological traits

#### Leaf gas exchange measurements

Gas exchange measurements were carried out for the estimation of net photosynthetic rate (Pn), stomatal conductance (gs), inter-cellular CO_2_ concentration (Ci), and transpiration rate (Tr) on leaf blades of evenly oriented and maximum light-exposed flag leaves in five plants for each treatment using an open IRGA LI-COR 6400 system (LI-6400, Li-Cor Inc. USA) at 9:00–11:00 h (local time). Photosynthetic measurements were performed under light saturated conditions (1000 mmol photon m^−2^ s^−1^ of PPFD), at 25 °C and 380 µmol mol^−1^ CO_2_.

#### Leaf relative water content

The relative water content was determined according to the standard method given by Barrs and Weatherly, 1962^50^.

4 $$ {\text{RWC}}\; (\% ) = \frac{{{\text{FW }}{-} {\text{DW}}}}{{{\text{TW}} {-} {\text{DW}} }} \times 100 $$

where FW is the sample fresh weight, DW is the sample dry weight, and TW is the sample turgid weight.

#### Membrane stability index

The membrane stability index (MSI) was measured by using a conductivity meter by following the method of Khanna-Chopra and Selote, 2007^51^. Leaf samples of 200 mg were thoroughly washed in double distilled water and placed in 10 mL distilled water with two sets. One set was heated for 30 min at 40 °C in a water bath and electrical conductivity was measured (C1). The second set was boiled for 10 min at 100 °C in a boiling water bath and electrical conductivity was measured (C2). The MSI was estimated by the equation given below:

5 $$ {\text{MSI}} = \left[ {1{-} \left( {\frac{{{\text{C}}1}}{{{\text{C}}2}}} \right)} \right] \times 100 $$

#### Measurement of chlorophyll and carotenoid contents

Frozen leaf samples (0.2 g) were placed for 24 h in a vial with 4 mL of dimethyl sulphoxide for pigment extraction to determine chlorophyll content according to Li et al. (2000). The absorbance of the supernatant was measured using a Pharmacia Ultra Spec Pro UV/VIS spectrophotometer (Pharmacia, Cambridge, England), at a wavelength of 663 nm, 645 nm, and 470 nm for chlorophyll *a*, *b*, and carotenoids respectively. The sum of chlorophyll *a* and *b* was used as total chlorophyll contents.

$$ \begin{gathered} {\text{Chlorophyll}}\;a \, = 12.7 \, \times {\text{ D}}_{663} - \, 2.69 \, \times {\text{ D}}_{645} \hfill \\ {\text{Chlorophyll}}\;b \, = 22.9 \, \times {\text{ D}}_{645} - \, 4.58 \, \times {\text{ D}}_{663} \hfill \\ {\text{Chlorophyll}}\;a \, + \, b \, = \, 20.2 \, \times {\text{ D}}_{645} + \, 8.02 \, \times {\text{ D}}_{663} \hfill \\ {\text{Carotenoids}} = \, (1000 \, \times {\text{ A}}_{470} - 3.27 \, \times {\text{ a}} - 104 \, \times {\text{ b}})/229 \hfill \\ \end{gathered} $$

### Statistical analysis

A three-way analysis of variance (ANOVA) was performed to determine the effect of treatments (three soil moisture levels, two cultivars and two PGPR treatments) on the investigated morphological as well as physiological parameters for each sampling and measurement point. Post hoc analysis was conducted using Duncan’s multiple comparison tests at P < 0.05 to define statistically significant differences between means for all variables of interest. All comparative analyses were conducted using the SPSS statistical package (SPSS Inc., Chicago, IL, USA). Figures were plotted by using Sigma Plot 10.0 software (Systat Software Inc., Chicago, IL, USA).

## References

[1] FAO (2015). Crop Prospects and Food Situation. Food and Agriculture Organization, Global Information and Early Warning System.

[2] Lefe I, Legay S, Lamoureux D (2017). Identification of drought-responsive compounds in potato through a combined transcriptomic and targeted metabolite approach. J. Exp. Bot..

[3] Monneveux P, Ramírez DA, Pino M (2013). Drought tolerance in potato. Can we learn from drought tolerance research in cereals?. Plant Sci..

[4] Nunes-nesi A, Fernie AR, Stitt M (2010). Metabolic and signaling aspects underpinning the regulation of plant carbon nitrogen interactions. Mol. Plant.

[5] FAO. How to Feed the World in 2050. Insights from an Expert Meet. *FAO 2050*, 1–35 (2009).

[6] Mauromicale G, Ierna A, Marchese M (2006). Chlorophyll fluorescence and chlorophyll content in field-grown potato as affected by nitrogen supply, genotype, and plant age. Photosynthetica.

[7] Wegener CB, Jansen G (2013). Antioxidants in different potato genotypes: effect of drought and wounding stress. Agriculture.

[8] Mahajan S, Mahajan S, Tuteja N, Tuteja N (2005). Cold, salinity and drought stresses: an overview. Arch. Biochem. Biophys..

[9] Schafleitner R, Gutierrez R, Legay S, Evers D, Bonierbale M (2007). Drought Stress Tolerance Traits of Potato.

[10] Zlatev Z, Lidon FC (2012). An overview on drought induced changes in plant growth, water relations and photosynthesis. Emirates J. Food Agric..

[11] Abid M, Ali S, Qi LK, Zahoor R, Tian Z, Jiang D, Snider JL, Dai T (2018). Physiological and biochemical changes during drought and recovery periods at tillering and jointing stages in wheat (*Triticum aestivum* L.). Sci. Rep..

[12] Gangadhar BH, Sajeesh K, Venkatesh J, Gene S (2016). Enhanced tolerance of transgenic potato plants over-expressing non-specific lipid transfer protein-1 (StnsLTP1) against multiple abiotic stresses isolation and gateway cloning of. Front. Plant Sci..

[13] Ji X, Shiran B, Wan J, Lewis DC, Jenkins CLD, Condon AG, Richards RA, Dolferus R (2010). Importance of pre-anthesis anther sink strength for maintenance of grain number during reproductive stage water stress in wheat. Plant Cell Environ..

[14] Powell N, Ji X, Ravash R, Edlington J, Dolferus R (2012). Yield stability for cereals in a changing climate. Funct. Plant Biol..

[15] Lulsdorf MM, Yuan HY, Slater SMH, Vandenberg A, Han X, Zaharia LI, Abrams SR (2013). Endogenous hormone profiles during early seed development of *C. arietinum* and *C. anatolicum*. Plant Growth Regul..

[16] Xu ZZ, Zhou GS (2006). Combined effects of water stress and high temperature on photosynthesis, nitrogen metabolism and lipid peroxidation of a perennial grass *Leymus chinensis*. Planta.

[17] Lee B, Farag MA, Park HB, Kloepper JW, Lee SH, Ryu CM (2012). Induced resistance by a long-chain bacterial volatile: elicitation of plant systemic defense by a C13 volatile produced by *Paenibacillus polymyxa*. PLoS ONE.

[18] Park Y-S, Dutta S, Ann M, Raaijmakers JM, Park K (2015). Promotion of plant growth by *Pseudomonas fluorescens* strain SS101 via novel volatile organic compounds. Biochem. Biophys. Res. Commun..

[19] Saharan BS, Nehra V (2011). Plant growth promoting rhizobacteria: a critical review. Life Sci. Med. Res..

[20] Zhang H, Kim MS, Krishnamachari V, Payton P, Sun Y, Grimson M, Farag MA, Ryu CM, Allen R, Melo IS, Paré PW (2007). Rhizobacterial volatile emissions regulate auxin homeostasis and cell expansion in Arabidopsis. Planta.

[21] Adesemoye AO, Egamberdieva D (2013). Beneficial Effects of Plant Growth-Promoting Rhizobacteria on Improved Crop Production. Prospects for Developing Economies.

[22] Glick BR (2005). Modulation of plant ethylene levels by the bacterial enzyme ACC deaminase. FEMS Microbiol. Lett..

[23] Meng S, Zhang C, Su L, Li Y, Zhao Z (2016). Nitrogen uptake and metabolism of *Populus simonii* in response to PEG-induced drought stress. Environ. Exp. Bot..

[24] Mmbaga GW, Mtei KM, Ndakidemi PA (2014). Extrapolations on the use of rhizobium inoculants supplemented with phosphorus (P) and potassium (K) on growth and nutrition of legumes. Agric. Sci..

[25] Hashem A, AbdAllah EF, Alqarawi AA, Aldubise A, Egamberdieva D (2015). Arbuscular mycorrhizal fungi enhances salinity tolerance of *Panicum turgidum* Forssk by altering photosynthetic and antioxidant pathways. J. Plant Interact..

[26] Sprenger H, Erban A, Seddig S, Rudack K, Thalhammer A, Le MQ, Kopka J, Hincha DK, Walther D, Zuther E, Karin IK (2018). Metabolite and transcript markers for the prediction of potato drought tolerance. Plant Biotechnol. J..

[27] Spitters CJT, Groot PJ (1989). Effects of water stress on photosynthesis and chlorophyll fluorescence of five potato cultivars. Potato Res..

[28] Gallé A, Haldimann P, Feller U (2007). Photosynthetic performance and water relations in young pubescent oak (*Quercus pubescens*) trees during drought stress and recovery. New Phytol..

[29] Beckers GJ, Conrath U (2007). Priming for stress resistance: from the lab to the field. Curr. Opin. Plant Biol..

[30] Ekin Z, Faruk O, Erman M, Erdal Ö (2009). The effect of *Bacillus* sp. OSU-142 inoculation at various levels of nitrogen fertilization on growth, tuber distribution and yield of potato (*Solanum**tuberosum* L.). Afr. J. Biotechnol..

[31] Galmés J, Medrano H, Flexas J (2007). Photosynthetic limitations in response to water stress and recovery in Mediterranean plants with different growth forms. New Phytol..

[32] Owen DW, Williams AP, Griffith GW, Withers P (2014). Use of commercial bio-inoculants to increase agricultural production through improved phosphorous acquisition. Appl. Soil Ecol..

[33] Abid M, Yuhang S, Liu S, Wang F, Gao J, Tian Z, Tingbo D (2017). Pre-drought priming sustains grain development under post-anthesis drought stress by regulating the growth hormones in winter wheat (*Triticum**aestivum* L.). Planta.

[34] Vile D, Garnier É, Shipley B, Laurent G, Navas ML, Roumet C, Lavorel S, Díaz S, Hodgson JG, Lloret F, Midgley GF, Poorter H, Rutherford MC, Wilson PJ, Wright IJ (2005). Specific leaf area and dry matter content estimate thickness in laminar leaves. Ann. Bot..

[35] Lizana C, Wentworth M, Martinez JP, Villegas D, Meneses R, Murchie EH, Pastenes C, Lercari B, Vernieri P, Horton P, Pinto M (2006). Differential adaptation of two varieties of common bean to abiotic stress I. Effects of drought on yield and photosynthesis. J. Exp. Bot..

[36] Abid M, Ali S, Qi LK, Zahoor R, Tian Z, Jiang D, Snider JL, Dai T (2018). Physiological and biochemical changes during drought and recovery periods at tillering and jointing stages in wheat (*Triticum aestivum* L.). Sci. Rep..

[37] Bieker S, Zentgraf U (2013). Plant senescence and nitrogen mobilization and signaling. Senescence Senescence Relat. Disord..

[38] Izanloo A, Condon AG, Langridge P, Tester M, Schnurbusch T (2008). Different mechanisms of adaptation to cyclic water stress in two South Australian bread wheat cultivars. J. Exp. Bot..

[39] Saneoka H, Moghaieb REA, Premachandra GS, Fujita K (2004). Nitrogen nutrition and water stress effects on cell membrane stability and leaf water relations in Agrostis palustris Huds. Environ. Exp. Bot..

[40] Banik P, Zeng W, Tai H, Bizimungu B, Tanino K (2016). Effects of drought acclimation on drought stress resistance in potato (*Solanum**tuberosum* L.) genotypes. Environ. Exp. Bot..

[41] Fischer RA (2008). The importance of grain or kernel number in wheat: a reply to Sinclair and Jamieson. Field Crops Res..

[42] Wu S, Hu C, Tan Q, Nie Z, Sun X (2014). Effects of molybdenum on water utilization, antioxidative defense system and osmotic-adjustment ability in winter wheat (*Triticum aestivum*) under drought stress. Plant Physiol. Biochem..

[43] Abid M, Tian Z, Ata-ul-karim ST, Liu Y, Cui Y, Zahoor R, Jiang D, Dai T (2016). Improved tolerance to post-anthesis drought stress by pre-drought priming at vegetative stages in drought-tolerant and -sensitive wheat cultivars. Plant Physiol. Biochem..

[44] Yemm EW, Willis AJ (1954). The estimation of carbohydrates in plant extracts by anthrone. Biochem. J..

[45] Roensen D, Johnson DB (1961). Estimation of protein in cellular material. Nature.

[46] Tang B, Xu S, Zou X, Zheng Y, Qiu F (2010). Changes of antioxidative enzymes and lipid peroxidation in leaves and roots of waterlogging-tolerant and waterlogging-sensitive maize genotypes at seedling stage. Agric. Sci. China.

[47] Zhang H (2012). Post-anthesis alternate wetting and moderate soil drying enhances activities of key enzymes in sucrose-to-starch conversion in inferior spikelets of rice. J. Exp. Bot..

[48] Barrs HD, Weatherly PE (1962). A re-examination of the relative turgidity technique for estimating water deficits in leaves. Aust. J. Biol. Sci..

[49] Khanna-Chopra R, Selote DS (2007). Acclimation to drought stress generates oxidative stress tolerance in drought-resistant than -susceptible wheat cultivar under field conditions. Environ. Exp. Bot..

[50] Tahir HAS, Gu Q, Wu H, Niu Y, Huo R, Gao X (2017). Bacillus volatiles adversely affect the physiology and ultra-structure of *Ralstonia solanacearum* and induce systemic resistance in tobacco against bacterial wilt. Sci. Rep..

[51] Tahir HAS, Gu Q, Wu H, Raza W, Hanif A, Wu L, Colman MV, Gao X (2017). Plant growth promotion by volatile organic compounds produced by *Bacillus subtilis* SYST2. Front. Microbiol..

